# A Negative Regulator of Cellulose Biosynthesis, *bcsR*, Affects Biofilm Formation, and Adhesion/Invasion Ability of *Cronobacter sakazakii*

**DOI:** 10.3389/fmicb.2017.01839

**Published:** 2017-09-26

**Authors:** Jian-xin Gao, Ping Li, Xin-jun Du, Zhong-hui Han, Rui Xue, Bin Liang, Shuo Wang

**Affiliations:** ^1^Key Laboratory of Food Nutrition and Safety, Ministry of Education, Tianjin University of Science and Technology, Tianjin, China; ^2^Beijing Advanced Innovation Center for Food Nutrition and Human Health, Beijing Technology and Business University, Beijing, China

**Keywords:** *bcsR*, *Cronobacter sakazakii*, cellulose biosynthesis, biofilm formation, adhesion/invasion

## Abstract

*Cronobacter sakazakii* is an important foodborne pathogen that causes neonatal meningitis and sepsis, with high mortality in neonates. However, very little information is available regarding the pathogenesis of *C. sakazakii* at the genetic level. In our previous study, a cellulose biosynthesis-related gene (*bcsR*) was shown to be involved in *C. sakazakii* adhesion/invasion into epithelial cells. In this study, the detailed functions of this gene were investigated using a gene knockout technique. A *bcsR* knockout mutant (Δ*bcsR*) of *C. sakazakii* ATCC BAA-894 showed decreased adhesion/invasion (3.9-fold) in human epithelial cell line HCT-8. Biofilm formation by the mutant was reduced to 50% of that exhibited by the wild-type (WT) strain. Raman spectrometry was used to detect variations in biofilm components caused by *bcsR* knockout, and certain components, including carotenoids, fatty acids, and amides, were significantly reduced. However, another biofilm component, cellulose, was increased in Δ*bcsR*, suggesting that *bcsR* negatively affects cellulose biosynthesis. This result was also verified via RT-PCR, which demonstrated up-regulation of five crucial cellulose synthesis genes (*bcsA, B, C, E, Q*) in Δ*bcsR*. Furthermore, the expression of other virulence or biofilm-related genes, including flagellar assembly genes (*fliA, C, D*) and toxicity-related genes (*ompA, ompX, hfq*), was studied. The expression of *fliC* and *ompA* in the Δ*bcsR* mutant was found to be remarkably reduced compared with that in the wild-type and the others were also affected excepted *ompX*. In summary, *bcsR* is a negative regulator of cellulose biosynthesis but positively regulates biofilm formation and the adhesion/invasion ability of *C. sakazakii*.

## Introduction

*Cronobacter sakazakii* is a Gram-negative, non-spore-forming, rod-shaped bacterium within the genus of *Cronobacter* spp., which is a group of emerging opportunistic pathogens associated with meningitis, septicemia, and necrotizing enterocolitis in neonates and infants. The mortality among infected infants is as high as 40–80% (Iversen and Forsythe, 2003). In addition, some patients who survive the disease suffer from mental or physical developmental delay and quadriplegia (Lai, 2001; Holy and Forsythe, 2014). *Cronobacter* spp. also infect elderly and immunocompromised adults. Unlike neonatal infection, *Cronobacter* is associated with bacteremia, osteomyelitis, splenic abscesses, pneumonia, conjunctivitis, wound infections, and urinary tract infections in adults (Blackwood and Hunter, 2016).

*C. sakazakii* has the capacity to adhere to human intestinal epithelial and brain microvascular endothelial cells (Quintero et al., 2011). The first step before colonization and infection for most pathogens is adherence to host cell surfaces. Bacterial variants that do not express adherence factors are unable to adhere and initiate infections; thus, adherence is important for pathogenicity (Cleary et al., 2004). Adherence to intestinal epithelial cells is a key step that determines whether an infection becomes refractory to treatment because *C. sakazakii* entries into intestinal epithelial cells is followed by entrance into the bloodstream, invasion of brain microvascular endothelial cells, and survival in the cerebrospinal fluid (Mange et al., 2006; Nair et al., 2009).

The ability of bacteria to attach to a surface and form biofilms facilitates the development of a continuous source of contamination and maintains disease incidence (Kives et al., 2006). A biofilm is generally defined as a structured community of bacterial cells that are adherent to a zoetic or abiotic surface and acts as an adhesive foundation and defense barrier (Aparna and Yadav, 2008). *Cronobacter* spp. are capable of attaching to different surfaces to form biofilms to resist multiple stress conditions, improve adherence, and increase pathogenesis (Lehner et al., 2005; Kim et al., 2006). Therefore, the investigation of biofilm characteristics, such as formation capacity and biochemical components, is helpful to better understand the mechanisms underlying *C. sakazakii* adherence, invasion of epithelial cells, and pathogenesis.

Some virulence factors involved in the interaction between *C. sakazakii* and host cells have been reported, including outer membrane protein A (OmpA) (Nair and Venkitanarayanan, 2007) and extracellular polysaccharides (EPS) (Iversen et al., 2004). However, very little information is available regarding *C. sakazakii* adhesion pathogenesis at the genetic level. In our previous work, we demonstrated the involvement of a cellulose production-related gene, *bcsR*, in interactions between *C. sakazakii* and intestinal epithelial cells using a random transposon insertion mutant library (Du et al., 2016). Cellulose (poly-β-(1 → 4)-D-glucose) is the most abundant biopolymer in nature. Most bacterial cellulose-producing genes encoding proteins involved in the UDP-glucose polymerization process are organized in a bacterial cellulose synthesis operon (*bcs*) (Wong et al., 1990; Recouvreux et al., 2008). The cellulose gene cluster, comprising 9 genes (*bcsCZBAQEFG* and *bcsR*), is present in nearly all *Cronobacter* strains (Ogrodzki and Forsythe, 2015). Among these 9 genes, *bcsR* is a short gene encoding a 62-amino-acid protein. Some studies have reported a role for *bcsR* in cellulose production in certain bacteria (Grimm et al., 2008; Serra et al., 2013). However, the role of this gene in the adhesion process is unclear.

In this study, we investigated the function of *bcsR* in *C. sakazakii* ATCC BAA-894 by constructing a gene mutant. The ability of the mutant to invade/adhere to HCT-8 cells was investigated and compared with that of the wild-type (WT) strain. Additionally, we assessed biofilm formation ability and performed Raman spectroscopy to analyze differences between the biochemical components in the WT and Δ*bcsR* mutant biofilms. This work helps to elucidate the role of *bcsR* in cellulose biosynthesis and *C. sakazakii* pathogenesis.

## Materials and methods

### Bacterial strains, plasmids, and growth conditions

All bacterial strains and plasmids used in this study are listed in Table 1. *C. sakaza*kii ATCC BAA-894 (WT and mutant strains) was routinely grown at 37°C in Tryptic Soy Broth (TSB; Difco, MD, USA) and on Tryptic Soy Agar (TSA; Difco, MD, USA) under aerobic conditions with constant shaking, unless indicated otherwise. When necessary, ampicillin, kanamycin, and chloramphenicol were used at 100, 100, and 10 μg/ml, respectively.

**Table 1:** Bacterial strains and plasmids used in this study.

**Strains or plasmids**	**Genotype or characteristics**	**Reference or source**
***Cronobacter sakazakii***		
ATCC BAA-894	WT	ATCC
*ΔbcsR*	*ΔbcsR*::km^r^	This study
*cpbcsR*	*ΔbcsR* with pACYC184-202	This study
***E. coli***		
DH5α	γ^−^Φ80d*lacZ*ΔM15Δ(*lacZYA-argF*)*U169 recA1 endA1 hsdR17*(${\text{r}}_{k}^{-}$ ${\text{m}}_{K}^{-}$) *supE44 thi-1 gyrA relA1*	38
**PLASMIDS**		
pKD46	*oriR101 repA101*(Ts) Amp^r^ *araADpgam-bet-exo*	39
pACYC184	p15A *ori* Cm^r^ Tet^r^	39
pACYC184-202	pACYC184 with *bcsR*	This study

### Construction of a *bcsR* gene deletion mutant

Site-specific mutation of *C. sakazakii* ATCC BAA-894 was performed using the Lambda-Red recombination method described by Geng et al. (2009). Briefly, the kanamycin resistance cassette from plasmid pET-26b was amplified using primers *kana*-F/*kana*-R. Two primer pairs, 202U-F/202U-R and 202D-F/202D-R, were used to amplify the upstream and downstream DNA sequences of the *bcsR* gene from the whole genome sequence of *C. sakazakii* ATCC BAA-894, obtained from GenBank (Grim et al., 2012). The amplified upstream and downstream DNA fragments of the *bcsR* gene and kanamycin resistance cassette were treated with appropriate restriction enzymes and subsequently cloned into the pMD18-T vector to produce pMD-*202* (*kana*), respectively, which was then transferred into *E. coli* DH5α. The segment consisting of *bcsR* upstream-*kana*-*bscR* downstream was cut from the vector and transformed into the wild-type (WT) strain *C. sakazakii* ATCC BAA-894 (harboring the pKD46 plasmid) via electroporation, and the kanamycin-resistant transformant, i.e., the *202::kana* mutant (*ΔbcsR*), was selected. All primer sequences are listed in Table 2.

**Table 2:** Primers used in this study.

**Gene amplified**	**Primers**	**Primer sequences (5′–3′)**	**Amplicon size (bp)**	**Note**
**MUTANT CONSTRUCTION**				
*kana*-F	Km^r^ cassette	CG*GGATCC*GAGGTATGTAGGCGGTGC	26	BamHI
*kana*-R		CGC*GTCGAC*ATATGTATCCGCTCATGAATT	30	SalI
202U-F	Upstream of EAS_04202	CG*GAATTC*CTTGCCTTACGGGTCATCTC	28	EcoRI
202U-R		CG*GGATCC*GGTTTATTTCCTGGCTTTCG	28	BamHI
202D-F	Downstream of EAS_04202	CGC*GTCGAC*CACAACGTGAACAACTCGC	28	SalI
202D-R		AA*CTGCAG*CCACGCGTAGGTTTCC	24	PstI
**COMPLEMENT-ATION**				
cp202-F	*bcsR* gene sequence	CG*GGATC*CGCACAGCAGCACAATGAAATAG	30	BamHI
cp202-R		CGC*GTCGAC*GCGCACGCCCTGTAATG	26	SalI
**qRT-PCR**				
Control-RT-F	16S rRNA	GAGTGGCGGACGGGTGAGT	19	
Control-RT-R		GTCCGTAGACGTTATGCGGTATTAG	25	
bcsQ-RT-F	*bcsQ*	GTCACGCCCGCTTACATCAG	20	
bcsQ-RT-R		TGCGTTTGCAGCCAGAGC	18	
bcsR-RT-F	*bcsR*	CTGAGAATGACGCTAAGGC	19	
bcsR-RT-R		CATCCTTGCGCTCCTG	16	
bcsE-RT-F	*bcsE*	TAATGAATGTCACCGGCAACTG	22	
bcsE-RT-R		GCCGACCAGCAAACTTCCAT	20	
bcsA-RT-F	*bcsA*	TGGTGTTGATCAACCTGCTCG	21	
bcsA-RT-R		CGCCGAGGATAATCAGGTTGTAG	23	
bcsB-RT-F	*bcsB*	CGCGACGATAAAGATTTACTCC	22	
bcsB-RT-R		GGTTTGACCTCGCCCACTT	19	
bcsC-RT-F	*bcsC*	TACGCCTACGGGCTTTATCTC	21	
bcsC-RT-R		TAGCCGTCTCCATCAGTTCATT	22	
fliA-RT-F	*fliA*	TGGCAGCGTTATGTCCCG	18	
fliA-RT-R		GCGTTCTACAGCATTCAGCAAG	22	
fliC-RT-F	*fliC*	CAAACGACACCAACGGTTCTACG	23	
fliC-RT-R		TGCCGTTGAAGTTAGCACCACC	22	
fliD-RT-F	*fliD*	CCGTCGCCCACGAAGTAG	18	
fliD-RT-R		GGCACAGCTCGGCATCAC	18	
ompA-RT-F	*ompA*	TCCAAAGGTATCCCGTCCAAC	21	
ompA-RT-R		GAGCAGCGCGAGGTTTCAC	19	
hfq-RT-F	*hfq*	GTCTCGTCCGGTTTCTCACCATAG	24	
hfq-RT-R		GAGAGGCAGCGGAAGATGGC	20	
ompX-RT-F	ompX	CATAGGAGAAGCCGTAGTCGC	21	
ompX-RT-R		GGCTTACCGTATCAATGACTGG	22	

### Complementation study

A complementation plasmid, containing the *bcsR*-coding sequence and its own promoter, was constructed. The *bcsR* gene was amplified by PCR using the primers 202 cp-F/202 cp-R (restriction sites were introduced into the primers) from *C. sakazakii* ATCC BAA-894 genomic DNA. The product was cloned into pACYC184 at appropriate restriction sites and then transferred into the mutant to generate Δ*bcsR* harboring pACYC184-202, *cpbcsR*. Nucleotide sequencing was performed to verify the sequence of the *bcsR*-coding region in the recombinant plasmid and complemented strain.

### Growth curves

*C. sakazakii* strain ATCC BAA-894 (WT) and the Δ*bcsR* and *cpbcsR* strains were cultured overnight in TSB medium at 37°C and subcultured as a 1% overnight culture in 100 ml of TSB medium. The cultures were incubated with shaking at 200 rpm per minute at 37°C for 14 h in a 100-ml conical flask. The optical density was measured at 600 nm (OD_600_) per hour.

### Scanning electron microscopy (SEM)

SEM was performed to examine the morphological differences between the *C. sakazakii* ATCC BAA-894 (WT), Δ*bcsR*, and *cpbcsR* strains (Wang et al., 2011). Bacterial strains were grown to the logarithmic phase and collected via centrifugation, and the pellets were fixed with 2.5% glutaraldehyde overnight at 4°C. The cells were washed three times with distilled water (5 min), followed by dehydration with an ethanol series (25, 50, 70, 80, 90, and 100%). The cells were further dried via vacuum freeze-drying for 1.5–2.5 h. The dehydrated bacterial powder was observed with a Hitachi SU1510 scanning electron microscope using an accelerating voltage of 5 kV (Hitachi, Tokyo, Japan).

### Adhesion/invasion assay

HCT-8 cells, derived from adenocarcinomas in a human colon and rectum (Tompkins et al., 1974; White et al., 1996), have been successfully used as an infection model to investigate pathogenesis of bacteria in previous studies (Luck et al., 2005; Pradel et al., 2015; Zargar et al., 2015). To evaluate bacterial invasion into HCT-8 cells (ATCC CCL-244; American Type Culture Collection, Manassas, Virginia), an invasion assay was conducted as described by Rogers et al. (2012), with modifications. Briefly, bacteria were incubated overnight aerobically at 37°C, transferred into fresh TSB medium as 1% inoculum and grown to 1 × 10^8^ CFU at 37°C with constant shaking. *C. sakazakii* cells were harvested by centrifugation at 3,000 g for 5 min, washed and resuspended in RPMI-1640 (Gibco, Invitrogen, USA). HCT-8 cells were grown in RPMI-1640 supplemented with 10% fetal bovine serum (FBS) (Gibco, Invitrogen, USA) in six-well tissue culture plates. The *C. sakazakii* cells were applied to the HCT-8 cell monolayer at a multiplicity of infection (MOI) of 100. After incubation for 1, 2, 3, or 4 h in the presence of 5% CO_2_, the cells were washed three times with PBS and lysed in 1 ml of 0.1% Triton X-100 for 10 min. The bacteria were serially diluted in PBS and plated on TSA for quantification.

### Analysis of biofilm formation capacity

The experiment was performed as previously described (Du et al., 2012), with modifications. *C. sakazakii* was inoculated into 5 ml of TSB and incubated at 37°C with aeration until the cell density reached 10^7^ CFU/ml. One hundred-microliter portions were loaded into a 96-well polystyrene plate and incubated at 37°C for 48 h without shaking. To fix the biofilms, 200 μl of 99% methanol was added for 15 min, the supernatants were removed, and the plates were air-dried. Subsequently, 200 μl of a 0.1% crystal violet (CV) solution was added. After 30 min, the excess CV was removed, and the plates were washed with normal saline (0.9% NaCl). Finally, the bound CV was released by adding 200 μl of 95% ethanol. The absorbance was measured at 570 nm using a Sunrise basic microplate reader (Tecan, Austria).

### Raman spectroscopy analysis

Raman spectroscopy analyses were performed to examine the biochemical profiles of *C. sakazakii* biofilms according to methods described in a previous report (Du et al., 2016), with modifications. Briefly, 100 μl of each logarithmic phase bacterial culture was dropped onto a Memberance Filter (Millipore, Ireland), and the filter was placed on a TSA plate containing appropriate antibiotics. The plates were incubated at 37°C for 72 h, and the media were changed every 24 h. Spectroscopic analyses were performed using a Renishaw inVia Raman system (Renishaw, Gloucestershire, UK) equipped with a 785-nm near-infrared diode laser and a Leica microscope (Leica Biosystems, Wetzlar, Germany). Raman spectra were collected using a WITec alpha300 Raman microscope (WITec, Ulm, Germany) equipped with a UHTS-300 spectrometer (Lu et al., 2011a; Wang et al., 2015). Wavenumbers from 400 to 1,800 cm^−1^ were selected for Raman-based chemometric analyses. Peaks were assigned based on methods described in a previous study (Du et al., 2016). The area and height of each Raman band were calculated using MATLAB, and significant (*P* < 0.05) band variations were characterized and analyzed. Twenty spectra were collected for each strain in triplicate at minimum. Principal component analysis (PCA) was performed to segregate the biofilms obtained from the WT, mutant, and complementation *C. sakazakii* strains by creating a two- or three-dimensional image (Lu et al., 2012).

### qRT-PCR

Quantitative RT-PCR assays were performed using SYBR® Premix Ex Taq II (Takara Bio Inc.), following the manufacturer's instructions, to investigate the transcriptional levels of various cellulose synthesis, biofilm-related, or virulence genes (Jing et al., 2016). Corresponding primers were designed based on the genome sequence of *C. sakazakii* ATCC BAA-894 (GenBank accession number CP000783.1; Table 2). Total bacterial RNA was isolated using an E.Z.N.A.™ Bacterial RNA Kit (Omega, USA). The 2^−ΔΔ*C*^_T_ value method was used to compare the expression of genes from different samples (Schmittgen and Livak, 2008). The 16S rRNA gene was used as an internal control for within-sample normalization of mRNA abundance (Choi et al., 2015). All real-time PCR reactions were performed using the Mastercycler ep gradient realplex system (Eppendorf, Germany). A reaction mixture lacking cDNA was used as the negative control.

Bacterial RNA was isolated from the WT strain (ATCC BAA-894), the *202* deletion mutant (Δ*bcsR*), or the complementation strain harboring the pACYC184-202 plasmid (*cpbcsR*). To obtain relative mRNA expression values on the y-axis, the mRNA level for each gene was divided by the mRNA level of the 16S rRNA-coding gene. The mRNA expression values were further normalized to the transcription levels exhibited by the WT strain. The means and standard deviations from three independent experiments are shown. Asterisks indicate significant differences (^*^ *P* < 0.05).

### Statistical analysis

Statistical analyses were carried out using Origin 8.0 (OriginLab Co., Northampton, MA) and MATLAB (MathWorks, Natick, MA, USA). Each experiment was independently repeated a minimum of three times to ensure reproducibility. All results were analyzed through the Duncan test and analysis of variance (ANOVA), a collection of statistical models used to analyze the differences among group means and their associated procedures (Tjur, 2005). The data are represented as the mean and standard deviation, and statistical analyses were performed using SPSS 19.0 (SPSS Inc., Chicago, IL, USA). Differences were considered statistically significant at *P* < 0.05.

## Results

### Construction and morphological characteristics of the Δ*bcsR* mutant in *C. sakazakii* ATCC BAA-894

**Figure 1:**
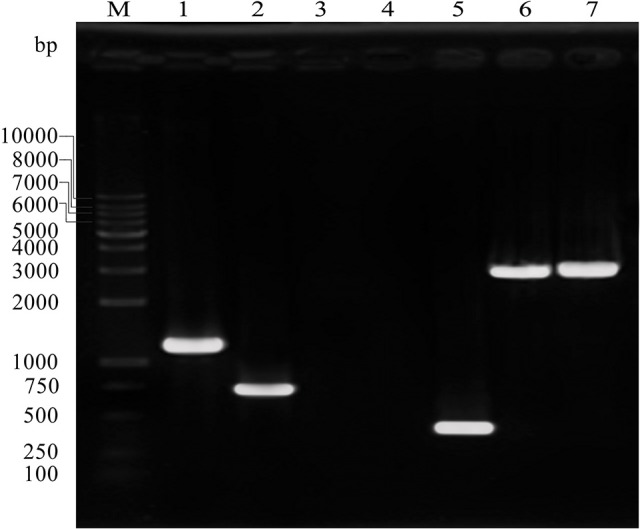
PCR verification of the Δ*bcsR* mutant strain using specific primers targeting sequences outside of homologous fragments and within the resistance gene. Sample 1, amplified with Kan F and KanR, using Δ*bcsR* as the template, 1,174 bp; sample 2, amplified with cp202-VF and cp202-VR, using *cp202* as the template, 738 bp; sample 3, amplified with 202F and 202R, using Δ*bcsR* as the template, 0 bp; sample 4, amplified with KanF and KanR, using WT as the template, 0 bp; sample 5, amplified with 202F and 202R, using WT as the template, 275 bp; sample 6, amplified with 202-VF and Kan-VR, using Δ*bcsR* as the template, 2,989 bp; sample 7, amplified with Kan-VF and 202-VR, using Δ*bcsR* as the template, 3047 bp; M, DL10000 marker. Electrophoresis was performed using 1% agarose.

The *C. sakazakii* gene (ESA_RS19400) homologous to the *bcsR* gene is located in a clockwise orientation in the genome of *C. sakazakii* ATCC BAA-894 and is also found in other *Cronobacter* spp. The gene (ESA_RS19400) exhibited high protein sequence similarity to the *bcsR* gene of *Kosakonia arachidis* (67%). However, the function of this gene is not yet known in *C. sakazakii*. To understand the roles of *bcsR* in pathogenesis of *C. sakazakii* ATCC BAA-894, we generated a mutant in which the entire *bcsR* gene was replaced by kanamycin resistance gene insertion using the λRed recombination technique. The Δ*bcsR* (*kana*) mutant was verified by PCR (Figure 1) and nucleotide sequencing. To further verify that the *bcsR* gene was knocked out, quantitative RT-PCR assays were performed, employing the bcsRY F/R primers and using WT, Δ*bcsR* or *cpbcsR* genomic DNA as the template. The results are shown in **Figure 6**. The expression of the *bcsR* gene was significantly reduced (26.57-fold change) in the Δ*bcsR* mutant when compared with that in the WT strain, which demonstrated that the *bcsR* gene was deleted.

**Figure 2:**
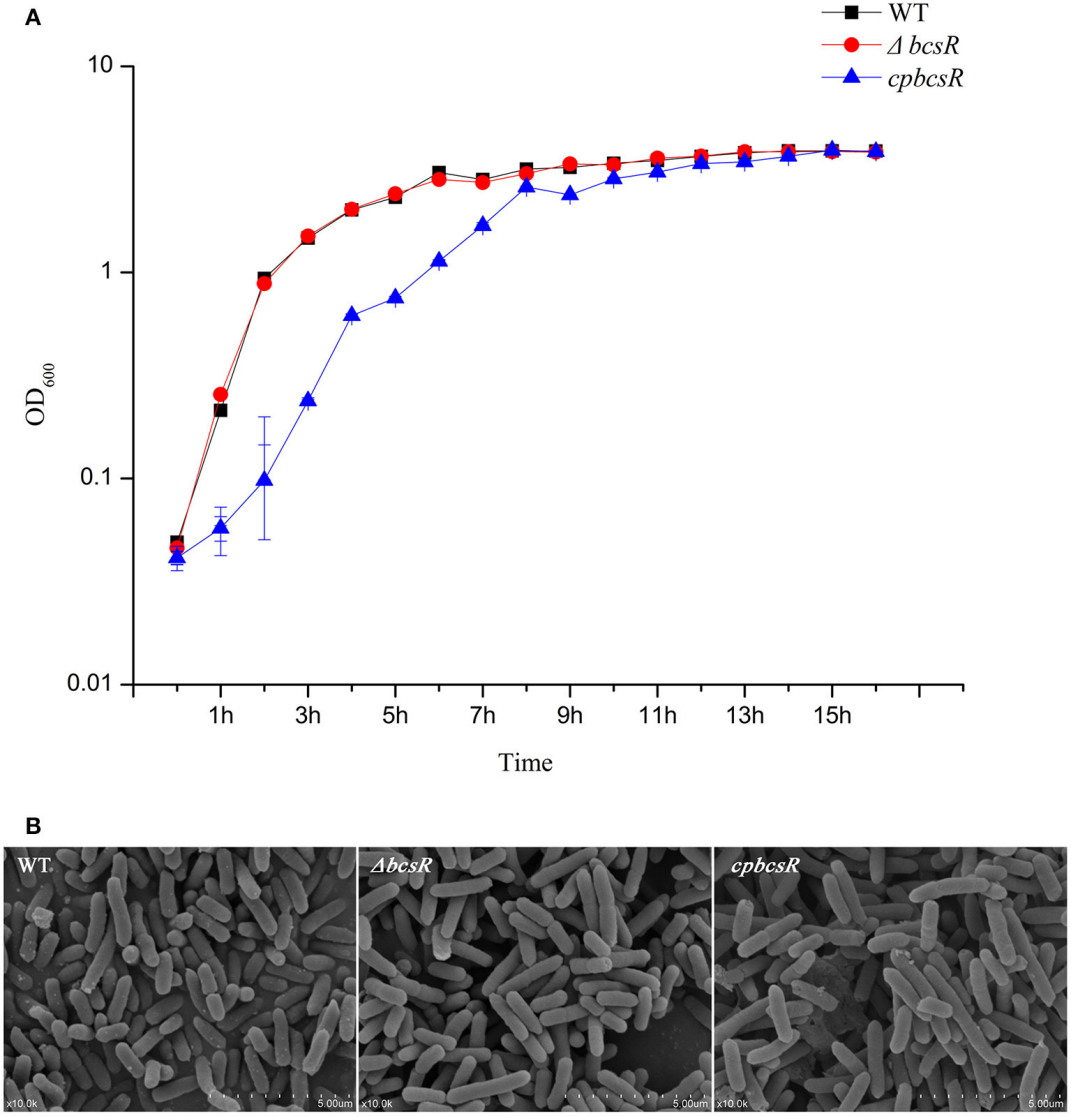
**(A)** Growth curves of *C. sakazakii* WT, Δ*bcsR*, and *cpbcsR* strains grown in the TSB broth at 37°C. The OD_600_ of each strain was measured hourly. Error bars represent the standard error of the mean (SEM) from three independent biological replicates. **(B)** SEM of biofilms formed on glass by WT and mutant strains. WT: WT ATCC BAA-894; Δ*bcsR*: mutant strain; *cpbcsR*: complementation strain.

The deletion mutant showed a similar growth rate to that of the WT strain (Figure 2A). However, the complementation strain (*cpbcsR*) demonstrated a slightly lower growth rate than that of the WT in the early stages of growth. In addition, similar morphological characteristics were observed in SEM images of the WT, Δ*bcsR*, and *cpbcsR* strains (Figure 2B). Thus, *bcsR* gene knockout did not affect bacterial growth and morphology in TSB medium.

### *bcsR* affects adhesion/invasion

**Figure 3:**
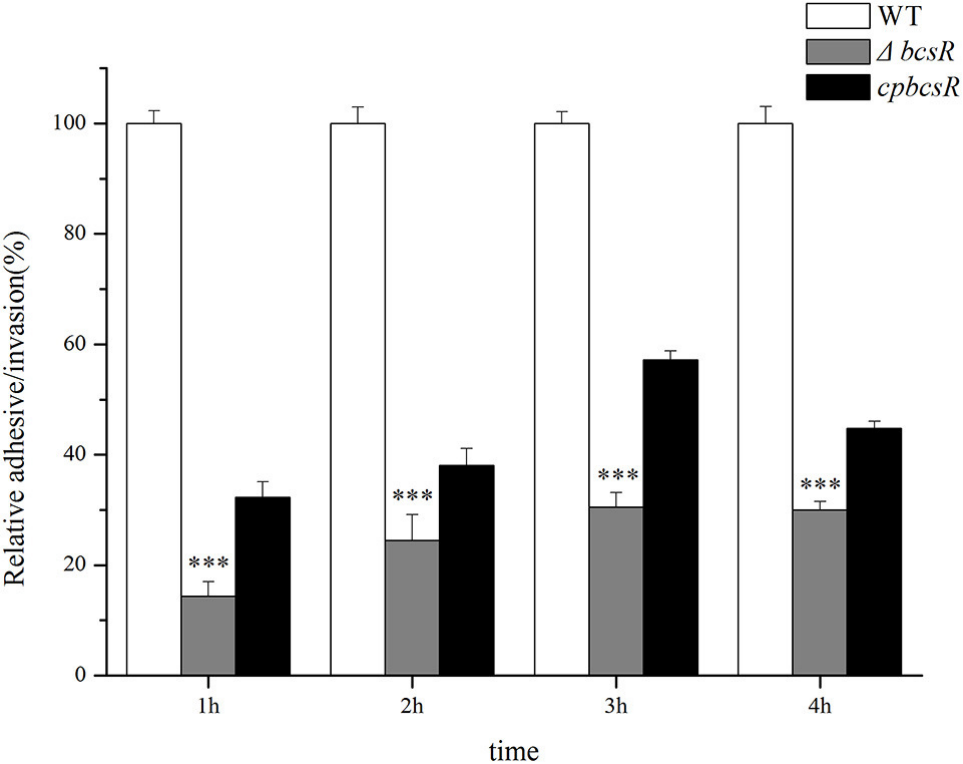
Absence of the *bcsR* gene impairs epithelial cell invasion at different times. Percent invasion for the mutant and complementation strains relative to the WT strain; ^***^p < 0.001.

In our previous work, we showed that the *bcsR* gene in *C. sakazakii* ATCC BAA-894 strain was involved in interactions with intestinal epithelial cells (Du et al., 2016). To explore the virulence-related functions of the *bcsR* gene in *C. sakazakii* ATCC BAA-894, an adhesion/invasion assay was performed at different time points (1, 2, 3, or 4 h) using HCT-8 cells (Figure 3). The invasion rate of the Δ*bcsR* mutant was significantly lower than that of the WT at 1, 2, 3, and 4 h in the invasion assay. Although the adhesion/invasion ability of the complementation strain did not reach that of the wild-type strain, it showed a significantly higher adhesion/invasion ability than the Δ*bcsR* mutant strain. The results suggested that the phenotypic defect of the Δ*bcsR* mutant was indeed attributable to the lack of *bcsR*, which led to a decreased adhesion/invasion ability in HTC-8 cells (Figure 3). Thus, we speculated that *bcsR* might positively regulate *C. sakazakii* adhesion/invasion in HCT-8 cells.

### Estimation of biofilm formation ability

**Figure 4:**
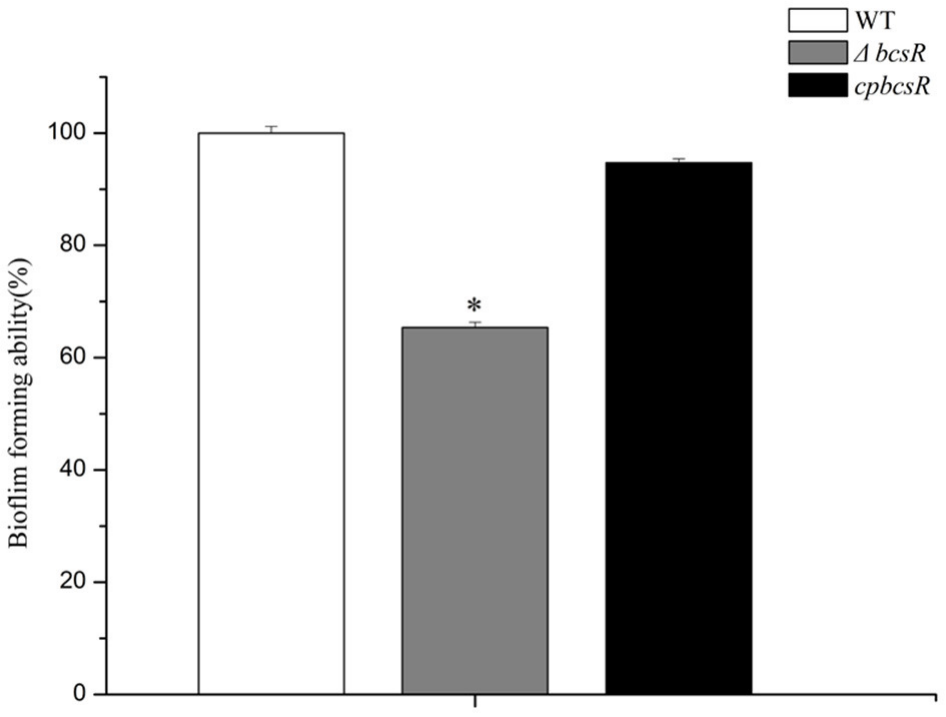
Biofilm formation by the WT, Δ*bcsR*, and *cpbcsR* strains. Percentages of biofilm formation for the mutant and complementation strains relative to the WT strain are shown. The error bars represent the mean standard deviation; ^*^P < 0.05.

To investigate whether the *bcsR* gene contributes to biofilm formation of *C. sakazakii* ATCC BAA-894, a CV staining assay was carried out. The Δ*bcsR* mutant showed a significant decrease (1.5-fold change) in biofilm formation compared with that in the WT, and the complementation strain demonstrated similar biofilm formation to that in the WT strain (Figure 4). The results suggest the *bcsR* gene is a positive effector in biofilm formation.

### Differences in biofilm composition between the *bcsR* mutant and WT strains

**Figure 5:**
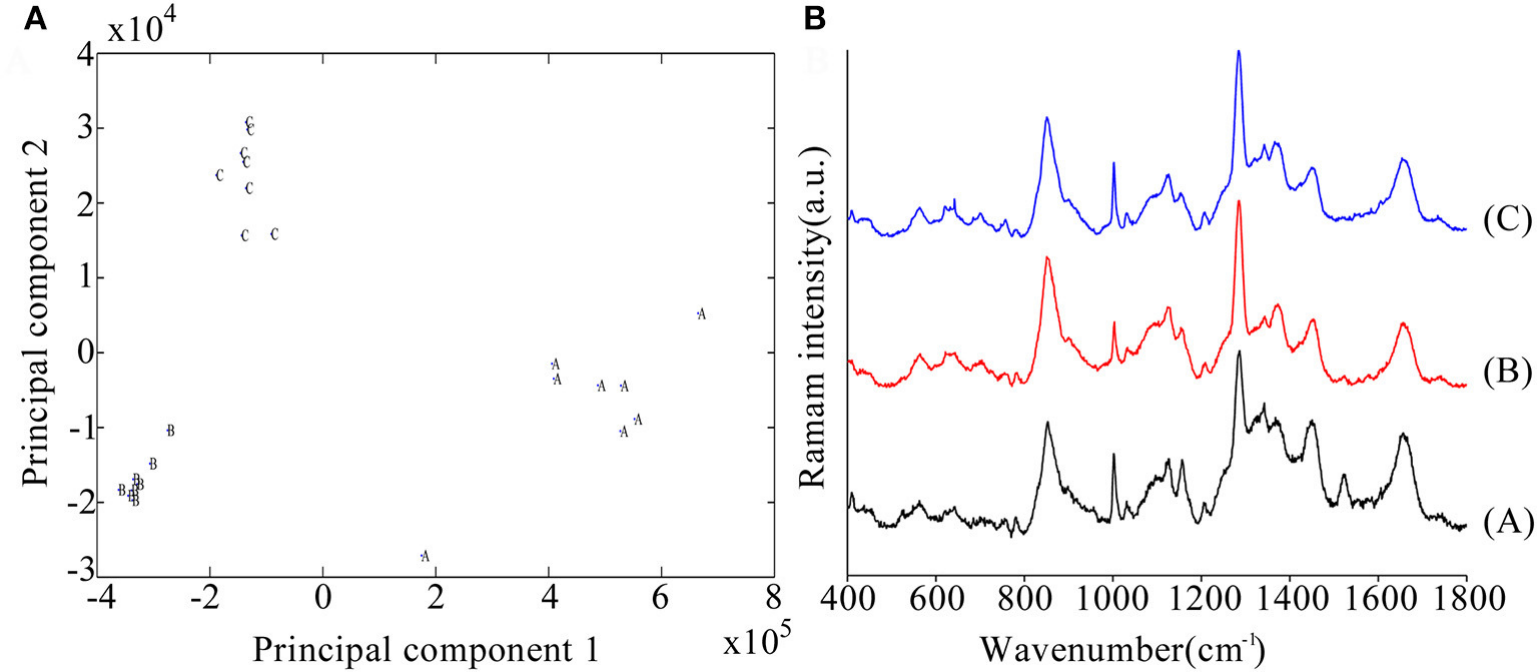
**(A)** Principal component analysis (PCA) model based on Raman spectral features. A: Wild-type strain, B: mutant strains, C: complementation strains. **(B)** Comparison of *C. sakazakii* biofilm spectral features between the WT (A), mutant (B), and complementation strains (C) using Raman spectroscopy.

To further investigate variations in the biochemical components of the biofilms formed by the tested strains, Raman spectroscopic analyses were performed. A PCA model was established to differentiate between the WT, mutant, and complementation strain. There were clear segregations between the WT, mutant, and complementation strains (Figure 5A), whose biochemical components exhibited variations.

A comparison of Raman spectra indicated that two peaks were higher (1,287 and 1,367 cm^−1^) and six peaks were lower (1,002, 1,157, 1,343, 1,450, 1,522, and 1,655 cm^−1^) in the mutant than in the WT strain, and these differences were significant (Table 3). The 1,287 and 1,367 cm^−1^ peaks were assigned to cytosine and cellulose respectively (De Gelder et al., 2007). The 1,002 cm^−1^ peak reflected the characteristics of phenylalanine (Movasaghi et al., 2007). The 1,157 and 1,522 cm^−1^ peaks were assigned to carotenoids (Feng et al., 2014). The 1,450 cm^−1^ peak was assigned to fatty acids (De Gelder et al., 2007). And the 1,655 cm^−1^ peak was assigned to amide I and amide III (Lu et al., 2011b).

**Table 3:** Raman intensities of different wavenumbers.

**Wave numbers**	**Raman intensity (a.u.)**		
	**WT**	***ΔbcsR***	***cpbcsR***
1002.84	2970.51a	1754.59b	2895.27a
1157.40	2759.70a	2396.31b	2230.74b
1287.64	3427.02a	6403.56b	5930.75b
1343.49	3464.45a	2982.50b	3168.39b
1367.63	2677.96a	4229.29b	3400.83c
1450.77	4399.85a	3326.01b	3033.83c
1522.21	2307.49a	473.93b	184.61c
1655.36	3875.54a	3470.61b	3375.95b

*“a,” “b,” and “c” in the same raw with different letters indicate significant difference at the 0.01 level according to one-way ANOVA, while the same letters indicate that the difference is not significant*.

To clearly express the differences between the wild-type, mutant, and complementation strains at various points, we present the Raman intensities at each Raman wavenumber in Table 3 and indicate the differences between each of the strains. The Figure S1 shows the distribution of Raman peaks.

### qRT-PCR analysis

The expression of several genes (*bcsR, bcsQ, bcsE, bcsA, bcsB, bcsC, fliA, fliC, fliD, ompA, hfq*, and *ompX*) involved in cellulose synthesis, flagella, and toxicity, was detected in the *bcsR* mutant and WT strain by qRT-PCR.

**Figure 6:**
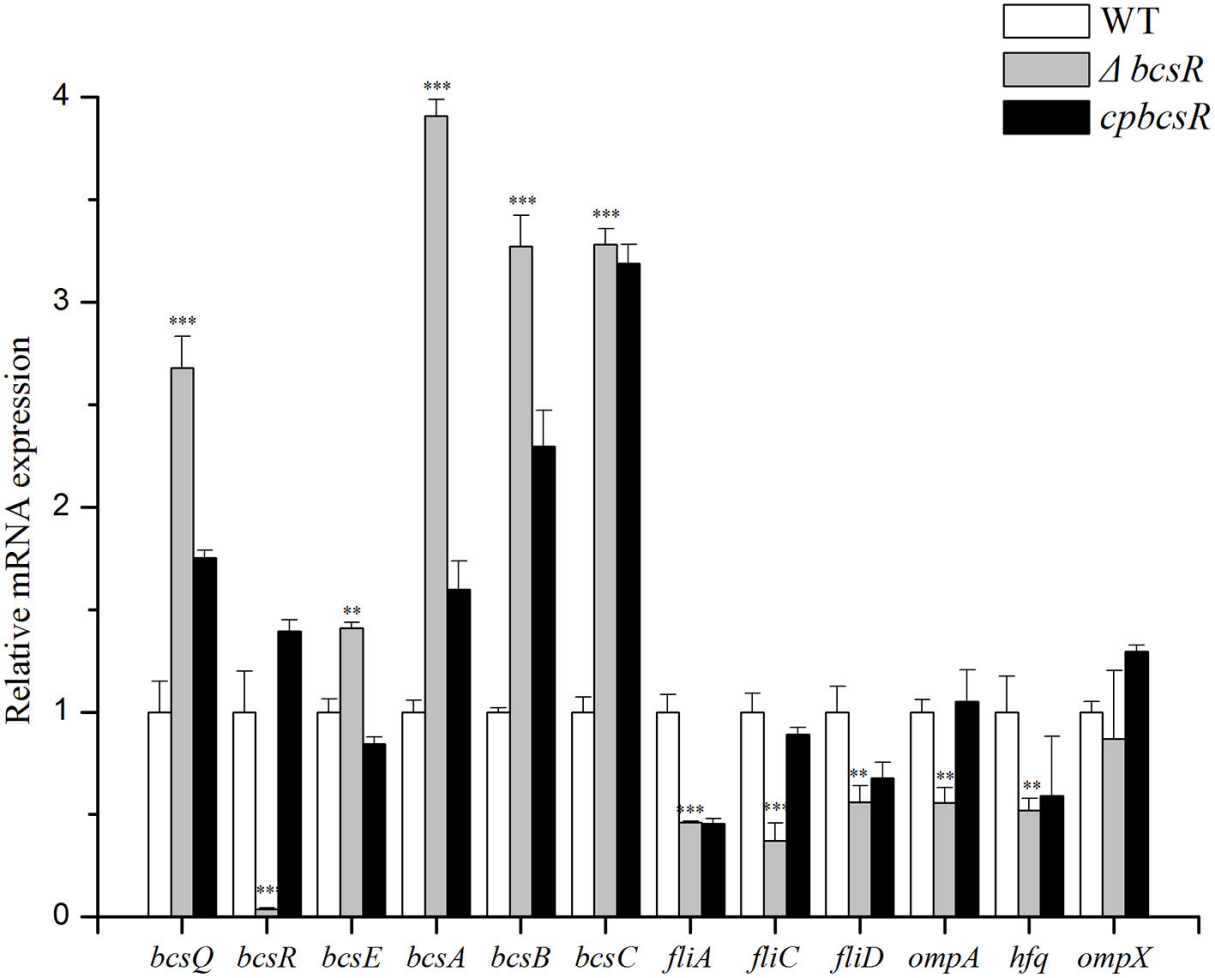
Transcription levels of 9 genes in *C. sakazakii* were determined by qRT-PCR. Experiments were repeated three times. The error bars represent the mean standard deviation; ^*^P < 0.05, ^**^P < 0.01, and ^***^P < 0.001.

The mRNA levels of the cellulose synthase operon genes *bcsQ, bcsE, bcsA, bcsB*, and *bcsC* increased by 2.68-, 1.41-, 3.91-, 3.27-, and 3.28-fold, respectively, in the Δ*bcsR* strain compared with those in the WT strain (Figure 6). The expression of *fliA, fliC*, and *fliD*, which are involved in regulating flagellar assembly, was reduced by 2.17-, 2.70-, and 1.79-fold, respectively, in the *bcsR* mutant compared with that in the WT strain (Figure 6). We also examined the expression levels of several toxicity-related genes, including *ompA, ompX*, and *hfq*, which have previously been associated with toxicity in *C. sakazakii*, in the *bcsR* mutant and WT strain. The mRNA levels of *ompA* and *hfq* genes in the Δ*bcsR* mutant decreased by 1.80- and 1.92-fold, respectively, compared with those in the WT strain (Figure 6). However, *ompX* transcription was not affected by the lack of *bcsR*. Thus, the *bcsR* gene down-regulated cellulosic synthesis in *C. sakazakii* ATCC BAA-894, in agreement with the Raman spectra data.

## Discussion

Bacterial pathogens must bind to epithelial cell surfaces prior to successful invasion. Therefore, adhesion and invasion into host tissue cells play essential roles in the virulence of most pathogenic bacteria. In our previous study, a cellulose biosynthesis-related gene was shown to be involved in *C. sakazakii* adhesion to epithelial cells. The *bcsR* gene was observed downstream of the *bcs* operon, which encodes enzymes or other proteins responsible for cellulose biosynthesis. However, the function of this gene in cellulose biosynthesis is unknown (Grimm et al., 2008). In this study, our data indicated that *bcsR* is a positive regulator of *C. sakazakii* adhesion/invasion into HCT-8 cells and biofilm formation.

Bacterial cells typically adhere to and interact with surfaces before eventually forming biofilms. Biofilms are defined as sessile communities embedded in polymeric substances produced by bacteria and are primarily composed of polysaccharides, proteins, and nucleic acids (Kim et al., 2006; Kolter and Greenberg, 2006; Serra et al., 2013). The ability to form a biofilm is a crucial trait that enhances bacterial resistance to environmental stresses and provides protection against drugs and disinfectants (Lehner et al., 2005; Furukawa et al., 2006). Acting as adhesive foundations and defense barriers, biofilms also play an important role in protecting embedded cells against detachment caused by flow shear. Potential bacterial toxicity may be enhanced when cells are present in biofilms, which are heterogeneously sessile bacterial communities that adhere to each other and to solid surfaces (Wang et al., 2012). Kunyanee et al. (2016) reported the important role of *Burkholderia pseudomallei* biofilms in bacterial pathogenesis in human epithelial cells with respect to initial attachment and invasion. In the Δ*bcsR* mutant of *C. sakazakii*, the decrease of biofilm formation suggests that this gene may affect adhesion/invasion by regulating biofilm synthesis.

According to the Raman spectra results, bands representing carotenoids, fatty acids and amides in the mutant exhibited significant decreases compared with those in the WT strain, indicating that *bcsR* may be positively associated with carotenoids, fatty acids and amides. The biofilms of certain *Cronobacter* strains contain high levels of carotenoids (Du et al., 2012). Therefore, *bcsR* may be positively associated with biofilm formation through the up-regulation of carotenoid, fatty acid, and amide biosynthesis in *C. sakazakii*.

The Raman spectra results also indicated that the band representing cellulose was greatly increased in the mutant compared with that in the WT strain, suggesting that *bcsR* may be a negative regulator of cellulose biosynthesis. Cellulose is an extracellular matrix component present in *C. sakazakii* biofilms (Grimm et al., 2008). Bacterial cellulose synthase is a multicomponent protein complex encoded in an operon containing at least three genes: *bcsA, bcsB*, and *bcsC* (Keiski et al., 2010). *bcsA, bcsB*, and *bcsC* encode for the cellulose synthase catalytic subunit, a cyclic-di-GMP binding protein and the cellulose oxidoreductase enzyme, respectively (Hu et al., 2015). The *bcsABC* genes in *Cronobacter* are necessary for cellulose production and are involved in biofilm formation and cell-cell aggregation (Hu et al., 2015). However, as a cellulose synthase operon gene, the function of *bcsR* remains even more elusive (Grimm et al., 2008). In this study, the decrease of biochemical components (including carotenoids, fatty acids, and amides) potentially explains the decline in biofilm formation after *bcsR* gene deletion in *C. sakazakii*. Interestingly, cellulose synthesis (Figure 5B) and cellulose synthase operon gene (*bcsABC*) expression levels significantly increased in the *bcsR* mutant (Figure 6). We hypothesize that *bcsR* affects the synthesis of other biofilm components during increased cellulose synthesis. In *Salmonella enteritidis*, the synthesis of colanic acid, lipopolysaccharide, enterobacterial common antigen, and cellulose are affected by the cellulose operon; these elements are highly important for biofilm formation (Solano et al., 2002). Cellulose is composed of β-D-1,4-glucan chains, and the precursor molecule is uridine diphosphate glucose (UDP-glucose) (Ross et al., 1991), which is also the substrate for the synthesis of colanic acid, lipopolysaccharide, and the enterobacterial common antigen. Excessive cellulose synthesis affects the synthesis of the other three components and thus affects biofilm synthesis (Solano et al., 2002). Channeling copious amounts of UDP-glucose to cellulose biosynthesis leads to the reprogramming of cellular metabolism to favor gluconeogenesis, which is a metabolic pathway that results in the generation of glucose from certain non-carbohydrate carbon substrates (Romling and Galperin, 2015). Furthermore, cellulose synthesis is energy-consuming and is affected by cellulose synthase activity. Cellulose synthase is specifically activated by the unique nucleotide cyclic diguanylic acid, which is synthesized from GTP by diguanylate cyclase. The synthesis of other biofilm components is also energy-consuming (Mika and Hengge, 2013). Therefore, excessive energy consumption for cellulose synthesis may not be conducive to the formation of other biofilm components. Consistent with our results, we speculate that excessive cellulose synthesis affects the synthesis of other components, thereby affecting biofilm formation.

Based on the qRT-PCR results, the expression of flagella (FliA, FliC, and FliD) and outer membrane proteins was reduced in the *bcsR* mutant. Therefore, we speculate that *bcsR* may be a global regulator of biofilm, flagella, and outer membrane protein biosynthesis, similar to CodY, which is a global transcriptional regulator that represses toxin gene expression by binding with high affinity to the *tcdR* promoter region (Girinathan et al., 2017). The *fliA, fliC*, and *fliD* genes regulate flagellar assembly (Chevance and Hughes, 2008). The *fliA* gene encodes an alternative sigma factor that regulates the transcription of class III flagellar genes, including filament structure genes and genes in the chemosensory pathway (Ohnishi et al., 1990; Chevance and Hughes, 2008; Choi et al., 2015). Because the *fliC* gene is a class III flagellar gene, and *fliD* encodes a late-secretion substrate of the filament-capping protein, free σ^28^ (a flagellar-specific sigma factor), which transcribes class III promoters, the enhanced expression of *fliC* and *fliD* may be attributable to large amounts of FliA, suggesting the *bcsR* gene affects the expression of class III flagellar genes via *fliA* (Chevance and Hughes, 2008). The three flagellar genes are reportedly involved in biofilm formation by *C. sakazakii* (Barken et al., 2008; Hartmann et al., 2010; Ye et al., 2016), consistent with our findings. Kim et al. (2010) reported important roles for OmpA and OmpX of *Cronobacter* in the invasion of Caco-2 and INT-407 cells. Hfq, oligomerized into a hexameric ring structure, is a posttranscriptional global regulator involved in the biosynthesis of OMPs, quorum sensing, stress responses, or metabolism and adhesin-mediating interactions with host tissue (Kakoschke et al., 2016). In our qTR-PCR analysis, the genes encoding OmpA, OmpX, and Hfq were all down-regulated in the mutant, suggesting their positive correlation with bacterial adhesion/invasion into epithelial cells. Based on the findings in this study, *bcsR* plays significant roles in biofilm formation and adhesion/invasion by regulating flagellar and toxicity-related genes.

Interestingly, in *bcsR* complementation strains, certain functions were restored, but *bcsR* expression levels only barely reached that of the wild strain. To explore whether this result was due to the gene polarity effects, we investigated the expression of genes flanking *bscR* in the WT, mutant, and complementation strains through qRT-PCR analysis (Figure 6). The expression of *bcsQ* and *bcsE* was investigated. *bcsQ* and *bcsE*, are the cellulose synthase operon genes that are closest to *bcsR* in the downstream region and upstream region, respectively. In the complemented strain, although the expression of the flanking genes was not recovered to wild-type levels, no significant difference was observed in the wild-type, mutant, and complemented strains (fold change <2). The lack of complete recovery of flanking gene expression may have been caused by the differences in the efficiency of *bcsR* gene expression in the complemented strain and the wild-type strain, considering that the *bcsR* gene was located in the pACYC184 plasmid in the complemented strain. This phenomenon was previously reported in studies performed by Kim et al. (2015) and Schilling and Gerischer (2009). Additionally, Kim et al. (2015) reported that failure of the pHFQ plasmid (a pACYC184 derivative with its own *hfq* promoter) to complement the hypermotility of the *hfq* strain might be caused by an imbalance of Hfq production from the low-copy-number pACYC184 plasmid. Therefore, we cannot completely exclude that the deletion of the *bcsR* gene did not affect the expression of other genes (*fliA, fliC, fliD, ompA*, and *hfq*) via polar effect. However, we are inclined to conclude that *bcsR* was a negative regulator for cellulose biosynthesis and we confirmed that cellulose biosynthesis was negatively related to biofilm formation and the epithelial cells adhesion/invasion capability of *C. sakazakii*.

## Conclusion

In this study, a gene knockout technique was employed to demonstrate the positive role of the *bcsR* gene in *C. sakazakii* adhesion/invasion in epithelial cells and biofilm formation. However, *bcsR* was found to act as a negative regulator of cellulose biosynthesis. Raman spectrometry and qRT-PCR results verified positive regulation of pathogenesis and negative regulation of cellulose biosynthesis by *bcsR* in *C. sakazakii*. This study significantly contributes to our understanding of the detailed functions of the *bcsR* gene in bacteria.

## Author contributions

JG contributed significantly to the conceived, designed, and performed the experiments, and the writing and editing of paper. PL, XD, and SW contributed significantly to conceived and designed of the work. ZH, RX, and BL mainly made a great contribution in performing the experiments. They all involved in the process of experimental design and the writing of paper. All authors agree to be accountable for the content of the work contributed to the conception of the study.

### Conflict of interest statement

The authors declare that the research was conducted in the absence of any commercial or financial relationships that could be construed as a potential conflict of interest.

## Supplementary material

Figure S1 Raman peak distribution of *C. sakazakii* biofilm.
