# Supplementary figures and images for: A Negative Regulator of Cellulose Biosynthesis, bcsR, Affects Biofilm Formation, and Adhesion/Invasion Ability of Cronobacter sakazakii
Source: Front Microbiol. 2017 Sep 26;8:1839. doi: 10.3389/fmicb.2017.01839 (PMC5649176; doi:10.3389/fmicb.2017.01839)

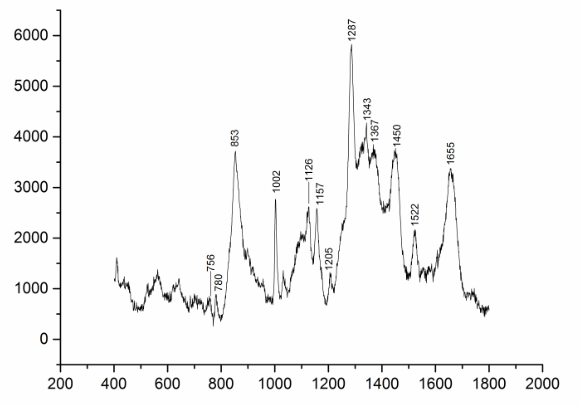

Supplement: Figure S1 — Raman peak distribution of C. sakazakii biofilm. [file Image1.PNG]
